# A bidirectional relationship between depression and the autoimmune disorders – New perspectives from the National Child Development Study

**DOI:** 10.1371/journal.pone.0173015

**Published:** 2017-03-06

**Authors:** Jack Euesden, Andrea Danese, Cathryn M. Lewis, Barbara Maughan

**Affiliations:** 1 MRC SGDP Centre, Institute of Psychiatry, Psychology and Neuroscience, King’s College London, London, United Kingdom; 2 Integrative Epidemiology Unit, University of Bristol, Bristol, United Kingdom; 3 Department of Child and Adolescent Psychiatry, Institute of Psychiatry, Psychology and Neuroscience, King's College London, London, United Kingdom; 4 Division of Genetics and Molecular Medicine, King’s College London, London, United Kingdom; Chiba Daigaku, JAPAN

## Abstract

**Background:**

Depression and the autoimmune disorders are comorbid—the two classes of disorders overlap in the same individuals at a higher frequency than chance. The immune system may influence the pathological processes underlying depression; understanding the origins of this comorbidity may contribute to dissecting the mechanisms underlying these disorders.

**Method:**

We used population cohort data from the 1958 British birth cohort study (the National Child Development Study) to investigate the ages at onset of depression and 23 autoimmune disorders. We used self-report data to ascertain life-time history of depression, autoimmune disorders and their ages at onset. We modelled the effect of depression onset on subsequent autoimmune disorder onset, and vice versa, and incorporated polygenic risk scores for depression and autoimmune disorder risk.

**Results:**

In our analytic sample of 8174 individuals, 315 reported ever being diagnosed with an autoimmune disorder (3.9%), 1499 reported ever experiencing depression (18.3%). There was significant comorbidity between depression and the autoimmune disorders (OR = 1.66, 95% CI = 1.27–2.15). Autoimmune disorder onset associated with increased subsequent hazard of depression onset (HR = 1.39, 95% CI = 1.11–1.74, *P* = 0.0037), independently of depression genetic risk. Finally, depression increased subsequent hazard of autoimmune disorder onset (HR = 1.40, 95% CI = 1.09–1.80, *P* = 0.0095), independently of autoimmune disorder genetic risk.

**Discussion:**

Our results point to a bidirectional relationship between depression and the autoimmune disorders. This suggests that shared risk factors may contribute to this relationship, including both common environmental exposures that increase baseline inflammation levels, and shared genetic factors.

## Introduction

An epidemiological link between psychiatric and autoimmune disorders has been observed for almost a century [1]. Despite this, the mechanism underlying this overlap is unclear, particularly in depression, one of the most common psychiatric disorders [2].

At this stage, most evidence in this area comes from relatively small-scale clinical studies exploring the association between depression and specific autoimmune disorders. In rheumatoid arthritis, multiple sclerosis and the inflammatory bowel diseases, authors have robustly demonstrated an increased overlap between depression and autoimmune disorder diagnosis in the same individuals, above that expected from their prevalences [3–5]. These relatively small clinical studies have been supplemented by two recent large-scale population-based studies. The first, on a Danish population, reported that depression is associated with a significantly increased risk of subsequent autoimmune disease (IRR = 1.25, 95% CI 1.19–1.31) [6]. The second, on a Taiwanese population, found a similar effect of depression on increased risk of subsequent rheumatoid arthritis (HR = 1.65, 95% CI = 1.41–1.77) [7].

In addition to any clinical implications, investigating the relationship between depression and autoimmune disorders, and identifying the factors driving it, will inform theories that the aetiology of depression involves immune processes [8,9]. In rheumatoid arthritis, low mood may predict subsequent worsening of symptoms in autoimmune disorder patients [10]. The aetiology and pathophysiology of depression is currently poorly understood, and current pharmacological treatments lack efficacy for mild to moderate depression [11]. It is therefore of great clinical importance to identify the mechanisms responsible for the onset of depression, and leverage this information in future work such as the repositioning of pharmaceuticals.

One approach to dissecting this relationship is to examine the relative ages at onset of the two disorders in order to infer elements of causality. If one disorder consistently precedes another, it may reliably increase risk of the second. Temporal precedence of this kind has long formed a criterion for establishing causality between two events, as first proposed by Hume and expanded on by others [12]. If there is no clear trend in the order of disorder onset, there are two possible interpretations. Firstly, a shared environmental risk factor may increase risk of both disorders. Secondly, the relationship may be due to pleiotropy—common genetic risk factors increasing risk of both disorders, as is seen between depression and a number of other comorbid psychiatric disorders such as schizophrenia [13].

As far as we are aware, only one study has explored relative ages at onset in depression and the autoimmune disorders to date. Using confirmed hospital diagnoses to identify cases, Andersson et al showed that depression elevated the hazard of autoimmune disorder onset in a Danish population cohort [6]. The use of hospital-confirmed diagnoses has the advantage of minimising ascertainment bias and any confounding effects of attrition. However, relying on date of first clinical contact as an indicator of age at onset may estimate an artificially late age at onset, as there is typically a latency between depression onset and clinical diagnosis [14]. Furthermore, this study did not include genetic information to clarify the origins of the comorbidity between depression and autoimmune disorders.

In the present study, we use data from the National Child Development Study (NCDS), a large epidemiological cohort comprised of all children born in England, Scotland and Wales, in one week of 1958. This sample has been followed up through their adult lives, providing data that allow the temporal analysis of onset of depression and autoimmune disorders. In addition, the availability of genetic data on NCDS cohort members enabled us to examine the contribution of genetic risk to disease onset, alongside traditional epidemiological methods. Risk of both depression and almost all autoimmune disorders studied to date including type 1 diabetes, rheumatoid arthritis, ankylosing spondylitis, Crohn's disease, psoriasis, primary sclerosing cholangitis and ulcerative colitis are influenced by large numbers of common polymorphisms (Single Nucleotide Polymorphisms, SNPs) of small effect, which often act to increase risk of a number of phenotypes concomitantly [15–19]. We therefore investigated the role of these common genetic risk factors within a longitudinal population cohort in order to dissect environmental and genetic risk factors influencing the relationship between depression and autoimmune disorders.

## Materials and methods

### Sample

We used data from the National Child Development Study [20], a sample comprising of all of the 17,638 individuals born in Scotland, England and Wales during one week of 1958. This cohort has been followed up on multiple occasions across childhood and in adulthood. We used self-report data from waves 5 (age 33), 6 (age 42) and 7 (age 46), collected in the years 1991, 2000 and 2004, along with genotype data derived from the biomedical survey undertaken in 2002–4, when cohort members were aged 44–45 years. Ethical approval for the medical examination and consent to obtain biomedical information was obtained by the 1958 British Birth Cohort team from South East MREC (ref: 01/1/44). Written consent was obtained from each participant to take part in the medical examination. This study is an analysis of previously collected data and therefore ethical approval was not required for this study.

### Measures

#### Autoimmune disorders

In wave 7 of the NCDS, participants were queried about their medical histories via telephone interview; responses were stored as ICD-10 codes alongside self-reported ages at onset. We investigated the following 23 autoimmune disorders, pooling them to form a single autoimmune disease phenotype: Addison's disease, autoimmune haemolytic anaemia, autoimmune thrombocytopenia purpura, celiac disease, dermatomyositis, Graves' disease, Hashimoto's thyroiditis, idiopathic myocarditis, idiopathic pulmonary fibrosis, insulin-dependent diabetes mellitus, inflammatory bowel disease (Crohn’s disease and ulcerative colitis), multiple sclerosis, myasthenia gravis, pernicious anaemia, polyarthritis, psoriasis, rheumatoid arthritis, scleroderma, Sjogren disease, systemic lupus erythematosus, vitiligo, and Wegener's granulomatosis. We pool disorders in order to increase power due to many autoimmune disorders being rare individually, and to mitigate any biases introduced through possible misclassification within the autoimmune disorders during interview. Participants were considered unexposed before autoimmune disease onset (or first autoimmune disease onset if a participant reported more than one), and exposed at age at onset and thereafter. All data were considered censored at age 46, the time-point of the most recent biomedical investigation.

#### Depression

We drew on three self-report measures of depression onset in the main analysis. In wave 5 (age 33), participants were asked if depression had ever been a problem, and if so at what age it was first a problem. In wave 6 (age 42), participants were asked the age they had started feeling depressed. Finally, in wave 7 (age 46), psychiatric histories were taken alongside age at onset (Table 1).

**Table 1:** Summary of depression metrics used to determine depression status and age at onset within the NCDS sample.

Wave	Age (year)	Depression Measure	Number of reported cases (% female)	Number of new cases (% female)
5	33 (1991)	Age at which depression was first a problem	390 (79.0%)	390 (79.0%)
6	42 (2000)	Age first started feeling depressed	1466 (66.1%)	1085 (61.5%)
7	46 (2004)	ICD codes for medical disorders and self reported age at onset. F32, 33 and 34	101 (62.3%)	24 (37.5%)

Participants were considered exposed from their earliest report of depression and exposed thereafter; as for the autoimmune disorders, reports of depression onset were censored at age 46. We took a number of steps to ensure the consistency in reports of age at onset across sweeps, and excluded any cases with inconsistent reports (S3 File).

### Genotyping

Blood samples and consent for genotyping were collected in the course of the biomedical survey. Genome-wide genotype data from a subset of NCDS participants were available from previous studies using five different genotyping chips. Quality control was performed on each chip separately: SNPs were removed based on MAF < 1%, Hardy Weinberg equilibrium *P <* 10^−5^, and missingness > 1%. Individuals with missingness > 10% were removed. In our phenotype-cleaned data set, 5762 participants were genotyped in total: 2896 participants were genotyped on the Illumina Immunochip, 1271 on the Illumina 1.2M chip, 1456 on the Infinium Humanhap, and 139 on the Affymetrix v6 chip.

### Polygenic Risk Scores—depression

Polygenic Risk Scores (PRS) for depression for each genotyped NCDS participant were calculated using genome-wide results from the Psychiatric Genomics Consortium MDD study [17]. PRS give a measure of genetic risk for each individual by summing the number of risk alleles carried, weighted by the natural logarithm of the odds ratio for each SNP as identified in GWAS. PRS were calculated using PRSice [21], including SNPs reaching *P*-value threshold of P_T_ = 0.3, previously shown to produce a reliable predictor of MDD in independent samples [17,22]. PRS were computed by genotyping chip and standardised to enable data to be pooled.

### Polygenic Risk Scores–rheumatoid arthritis

Of publically available GWAS results for autoimmune disorders, rheumatoid arthritis (RA) is one of the highest powered by both sample size—29,880 RA cases and 73,758 controls—and the number of genome-wide significant variants identified (n = 101) [18]. The NCDS contributed 1,999 controls to the WTCCC study [19], which is part of the RA GWAS meta-analysis; we therefore removed the effect of these samples from the Okada GWAS summary statistics by performing an association study on the WTCCC rheumatoid arthritis cases and controls, and performed an inverse meta-analysis in order to obtain a GWAS summary statistics which can be used to calculate PRS in the NCDS–this cleaning of GWAS data is outlined in detail below (S4 File).

The summary results from this adjusted RA GWAS meta-analysis were used to construct rheumatoid arthritis PRS for all genotyped NCDS individuals. To obtain the optimum SNP *P*-value threshold, we identified all NCDS rheumatoid arthritis and polyarthritis cases, and classified others as controls. We tested the ability of rheumatoid arthritis PRS to predict this case-control arthritis status at a number of SNP *P*-value thresholds. Logistic regression for case-control status was performed for each chip separately, and the PRS regression coefficient was meta-analysed across chips at each *P*-value threshold. This showed that the most predictive threshold was P_T_ = 0.001, consistent with the high power of the RA GWAS meta-analysis [23]. RA PRS at this *P*-value threshold were standardised by chip and used in modelling below.

### Statistical analyses

All analysis, unless stated otherwise, was performed using R version 3.2.2 [24]. We fitted Cox Proportional Hazards models to investigate the time-course of depression on autoimmune disorder onset and vice-versa, using Breslow’s method to estimate the baseline hazard function. In each case, the predictor variable is coded as a time-varying covariate; this is achieved by specifying multiple observations per individual, one before any exposure, one before any outcomes and a third thereafter. Individuals were considered unexposed the year before reported age at onset for a phenotype and exposed thereafter. For example in testing for the effect of depression on age at onset of autoimmune disorder, a participant reporting depression onset at age 20 and an autoimmune disorder at age 40 would be considered unexposed for depression until age 20, exposed for depression but not autoimmune disorder up to age 40, and then exposed for both depression and autoimmune disorder until age 46, the most recent point at which data was collected. Models were fitted using autoimmune disorder as the event, coding by exposure to depression at different time points, and similarly using depression as the event, coding for diagnosis of autoimmune disorder. All models were adjusted for gender as this is a strong predictor of both depression [25] and autoimmune disorder [26]. For each model, we tested the null hypothesis that the exposure (depression, autoimmune disorder) had no effect on the event (autoimmune disorder, depression). Finally, PRS for MDD were incorporated into the Cox Proportional Hazards models in order to estimate the genetic contribution to hazard of depression–firstly in an unadjusted model investigating hazard of depression and secondly in a model adjusting for any effect of autoimmune disorder onset; we then incorporate the same procedure to test the effect of PRS for rheumatoid arthritis predicting hazard of autoimmune disorder onset, extending this to a case where the onset of depression is adjusted for.

## Results

### Sample characteristics and overlap between autoimmune disorders and depression

After harmonisation across time-points and data cleaning, 8174 individuals (48% female) remained in our analytic sample. By age 46, 315 (3.85%; 55.6% female, Table 2) reported ever being diagnosed with an autoimmune disorder (an event-rate per 10,000 person-years of 8.38), and 1499 (18.3%; 65.6% female) were positive for our measure of depression. At age 46, 3 individuals report ever being diagnosed with bipolar disorder, 6 individuals report ever being diagnosed with schizophrenia. Of 6 participants reporting age at onset for autoimmune disorders before age 10, 4 report celiac disease and two report polyarthritis.

**Table 2:** Sample characteristics. Measures are presented separately for the full sample, and the subsample that have been genotyped–these are the participants used in the Polygenic Risk Score analysis. All controls are censored at age 46, so case status denotes onset before this age, and age at onset is within individuals who have onset before this age.

Measure	Full Sample	Genotyped Subsample
Sample Size	8174	5762
Female (%)	3919 (47.9%)	2902 (50.4%)
Number depressed (%)	1499 (18.3%)	1067 (18.5%)
Number (%) depression cases Female	984 (65.6%)	736 (69%)
Average depression age at onset (SD)	34.4 (6.3)	34.4 (6.36)
Average depression age at onset in women (SD)	33.9 (6.54)	33.8 (6.62)
Number with an autoimmune disorder (%)	315 (3.85%)	226 (3.92%)
Number (%) autoimmune disorder cases female	175 (55.6%)	131 (58%)
Average autoimmune disorder age at onset (SD)	33.2 (10.9)	33.5 (11)
Average autoimmune disorder age at onset in women (SD)	34.1 (10.4)	34.4 (10)

Depression and autoimmune disorders co-occurred in the same participants at a higher rate than would be expected by chance (84 individuals, Odds Ratio = 1.66, 95% CI = 1.27–2.15, Fisher’s exact test *P* = 1.92 x 10^−4^).

### Dissecting directionality via relative ages at onset

**Fig 1:**
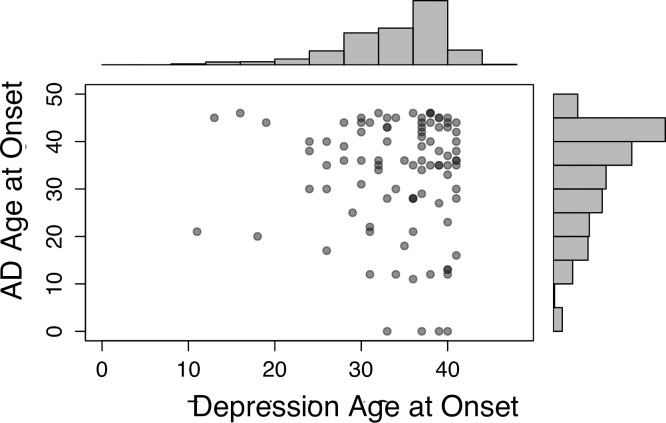
Age at onset distributions for depression and any autoimmune disorder (AD). Participants with both disorders are shown as points, with darker points representing more individuals with this pair of ages at onset.

The mean reported age at onset for autoimmune disorders was 33.2 years (SD = 10.9), and for depression was 34.4 years (SD = 6.3). Reported ages at onset were not significantly different for men and women for autoimmune disorders (*P* = 0.105), and were significantly later in males than females in depression (*P* = 6.93x10^-6^). The ages at onset of depression and the autoimmune disorders are shown on histograms in Fig 1, on the x and y axes respectively; ages at onset in individuals with both disorders are shown as points, with darker points indicating multiple individuals with the same combination of ages at onset.

**Fig 2:**
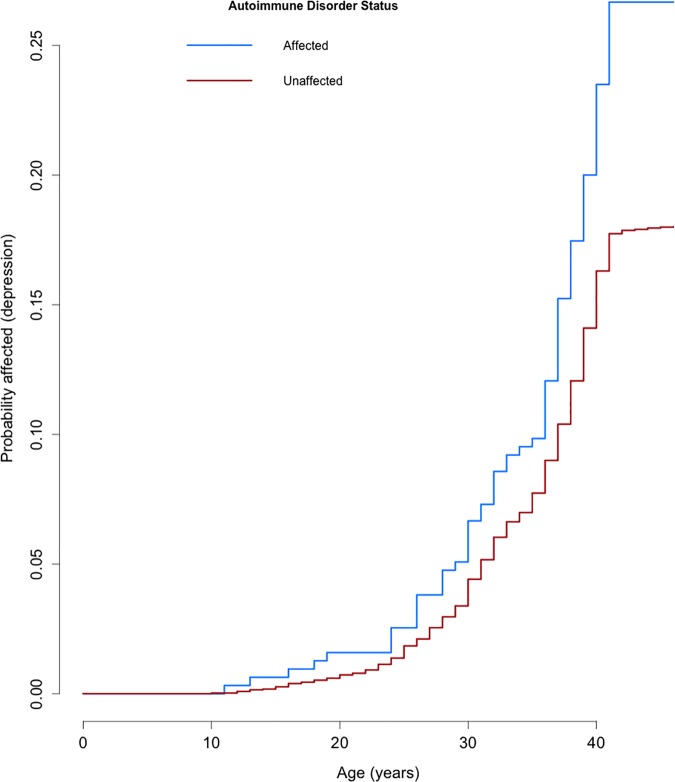
Curve illustrating age of onset of depression by reported autoimmune disorder status. These are Kaplan-Meier curves modified to show 1 minus survival

**Fig 3:**
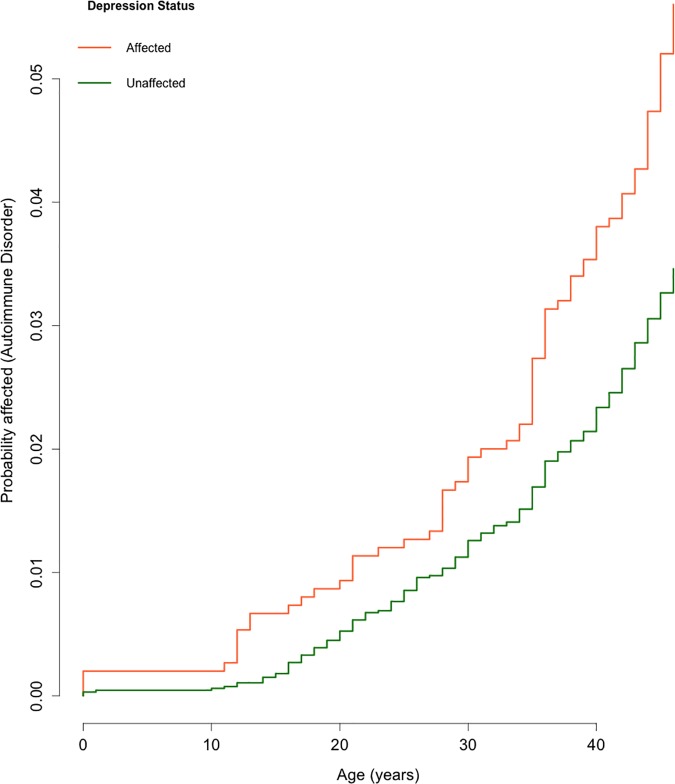
Curve illustrating age at onset for autoimmune disorders by reported depression status. These are Kaplan-Meier curves modified to show 1 minus survival

Cox Proportional Hazards models allow us to explore time-dependent changes in hazard, and to use time-varying covariates. We fitted Cox Proportional Hazards models treating autoimmune disorder as a time-varying covariate for depression onset, and depression onset as a time-dependent covariate for autoimmune disorder onset. Autoimmune disorder onset increased the hazard of subsequent depression onset, with a Hazard Ratio of 1.39 (95% CI = 1.11–1.74, *P* = 3.7 x 10^−3^), adjusting for gender. We also found evidence for an effect of depression onset increasing subsequent hazard of autoimmune disorder onset (HR = 1.40, 95% CI = 1.09–1.80, *P* = 9.5x10^-3^), adjusting for gender. These results are displayed graphically as Kaplan-Meier curves (Figs 2 and 3).

### Shared genetic risk

To test for shared risk genes increasing hazard of both depression and the autoimmune disorders, we incorporated PRS from genotype data on subset of the NCDS sample (N = 5762). As individuals were genotyped on one of 5 chips, we verified that standardised Polygenic Risk Scores (PRS) for depression did not differ across chips (ANOVA F = 0.57, *P*–value = 0.69). In an unadjusted model, MDD PRS was a significant predictor of depression hazard (HR = 1.09, 95% CI = 1.03–1.12, *P* = 5.3 x 10^−3^), and after adjusting for gender (HR = 1.08, 95% CI = 1.02–1.15, *P* = 9.1 x 10^−3^).

Including autoimmune disorder onset and MDD PRS in a Cox model adjusting for gender, we found that both MDD PRS (HR = 1.08, 95% CI = 1.02–1.15, *P* = 8.7 x 10^−3^) and autoimmune disorder onset (HR = 1.32, 95% CI = 1.01–1.72, *P* = 0.046) were significant, independent predictors of subsequent depression onset. Although the smaller Ns in the genotyped sample reduced the statistical power of this model, the point estimate for the Hazard Ratio of autoimmune disorder status on depression onset was closely similar to that in the phenotypic analysis reported above.

For RA PRS, we confirmed that scores did not differ by genotype chip (ANOVA F = 0.93, *P*-value = 0.45), and that scores predicted rheumatoid arthritis and polyarthritis case-control status (*P* = 0.005). In a Cox Proportional Hazards model adjusting for gender and depression onset, RA PRS predicted autoimmune disorder hazard (HR = 1.15, 95% CI = 1.01–1.31, *P* = 0.03), with a nominally significant independent effect of depression status (HR = 1.31, 95% CI = 0.97–1.38, *P* = 0.08).

We finally tested whether RA PRS predicted depression onset, and whether MDD PRS predicted AD onset. Neither of these associations were significant: HR = 0.99 (*P* = 0.79) and HR = 0.97 (*P* = 0.68) respectively. We repeated all of the above analyses using cluster robust standard errors in order to account for the effect of longitudinal dependence across observations. The results of these analyses confirm that accounting for the effects of this dependence has little effect on our results (S5 File).

## Discussion

We explored the relationship between depression and autoimmune disorders up to mid-life using epidemiological and genetic data. We replicated the finding that autoimmune disorders are frequently comorbid with depression, using a longitudinal national birth cohort and self-report data, which is increasingly used in the study of depression [27]. We also demonstrated an effect of autoimmune disorder onset increasing hazard of subsequent depression onset; this effect was broadly independent of the effect of genetic risk factors influencing hazard of depression. These results highlight the utility of a longitudinal approach to problems of medical comorbidities in epidemiology; our epidemiological results replicate those of Andersson et al [6],and build on them by including individuals who had not sought specialist mental health care, but who had explicitly answered questionnaires on history and current status of depression, within a population-based sample. There are a number of alternative explanatory models for the observed findings

### Causative effect of depression on autoimmune disorders

Longitudinal studies have shown that depression shows a two-way association with systemic inflammation [28], which is a key component in the pathophysiology of autoimmune disorders, such as rheumatoid arthritis [29]. Our finding that depression onset can increase subsequent hazard of autoimmune disorder onset is consistent with this model [30].

### Causative effect of autoimmune disorders on depression

The role of immune abnormalities in the pathophysiology of depression has been the focus of intense research for the past two decades [31]. For example, experimental stimulation with pro-inflammatory cytokines and bacterial compounds, such as lipopolysaccharides (LPS), induces a cluster of symptoms overlapping with depressive symptoms in animal models and humans [32,33]. Furthermore, anti-inflammatory medications appear to have antidepressant effects [34]. Our finding that autoimmune disorder onset can increase subsequent hazard of depression onset is consistent with these findings.

### Shared environmental factors

Shared environmental risk factors for both MDD and autoimmune disorders may confound interpretation of our results; previous literature suggests that childhood maltreatment but not cigarette smoking may be confounders. Temporal ordering–e.g. via looking at risk factors in childhood—may provide one route to differentiate between shared risk factors, and risk factors caused by a particular phenotype. There is robust evidence for an association between childhood maltreatment and both depression and the autoimmune diseases [35,36] and immune abnormalities appear concentrated within a subgroup of depressed individuals with a history of childhood maltreatment [37,38]. Therefore, early life stressors may play a role in the comorbidity of these two outcomes. Evidence for cigarette smoking as a confounder to our analysis is weaker–although cigarette smoking is associated with both MDD [39] and also autoimmune disorders such as rheumatoid arthritis [40], the association with MDD is unlikely to be causal. Whereas there is strong evidence that smoking increases risk of RA [41], there is considerable debate as to the direction of any causal association between smoking and MDD, with Bjorngaard et al finding no evidence for a causal effect of cigarette smoking in MDD using a Mendelian randomisation paradigm [39]. Whilst negative results in Mendelian randomisation studies are difficult to interpret, this supports the assertion that cigarette smoking is not confounding our analyses here.

### Shared genetic factors

Inflammatory models of MDD would suggest a predisposition to higher inflammatory activity, similar to the inflammatory arthritis that precedes rheumatoid arthritis, in depression patients [8]. Genetic risk of inflammatory over-activity may underlie the epidemiological relationship between these two families of disorders, however this conclusion would not be consistent with our results, as we found no evidence for genetic risk of rheumatoid arthritis increasing hazard of depression. Instead, we find evidence for independent effects of MDD genetic risk and autoimmune disorder onset on hazard of subsequent depression. Although data on family history of autoimmune or psychiatric disorders was not collected in this cohort, taking genetic risk as a proxy for family history demonstrates consistency with previous literature. An analysis of the Danish population register demonstrated a significant incidence rate ratio for schizophrenia amongst autoimmune disorder cases when stratifying by individuals with no family history of psychiatric disorders (IRR = 1.26, 95% CI = 1.11–1.43), but no evidence of such a relationship when stratifying by individuals with a family history of psychiatric disorders (IRR = 1.16, 95% CI = 0.93–1.44) [42].

We note that two separate causal mechanisms, one by which depression increases hazard of autoimmune disease and another by which autoimmune disease onset increases hazard of depression is a less parsimonious conclusion than the presence of a shared environmental risk factor.

### Clinical implications

Because depression can exacerbate systemic inflammation [28] and symptoms of autoimmune disorders [30], assessment and treatment of depression in patients with autoimmune disorders is crucial. In addition, not all patients with depression benefit from currently available treatments [11,36], and new treatments targeting patient subgroups with identified vulnerabilities, such as immune abnormalities, could offer innovative, effective strategies [9,34]. Finally, further investigations of shared pathways that may drive the observed comorbidity, including the roles of childhood maltreatment, can uncover key biological mechanisms [43].

### Limitations

Our findings need to be viewed in light of several limitations. First, the low prevalence of the individual autoimmune disorders—although consistent with rates expected in community samples—required that we group them together into a single composite phenotype. Whilst this has the advantage of mitigating confounding due to misclassification within the autoimmune disorders and provides increased power for modelling, this pooled autoimmune disorder phenotype prevented us detecting aetiologically distinct subgroups within autoimmune disorders. It also precluded our investigating the role of the genetics of autoimmune disorders in depression onset, beyond the marker for genetic risk of rheumatoid arthritis used in the present study.

We are also unable to investigate the effects of other psychiatric disorders such as schizophrenia and bipolar disorder on autoimmune disorder risk in this data set, due to two limitations. Firstly, the small sample sizes of participants diagnosed with schizophrenia and bipolar disorder– 6 and 3 respectively–would cause a substantial power concern. This low sample size would be expected due to the expected high drop-out amongst schizophrenia patients in cohort studies [44]. Secondly, we are unable to calculate polygenic risk scores for schizophrenia or bipolar disorder genetic risk, as the most recent Genome-Wide Association Studies for these disorders [13] incorporate the NCDS individuals as unscreened population controls.

A further limitation arising from our pooled autoimmune disorder phenotype is the difficulty in comparing reported prevalences by disorder. The event-rate for first autoimmune disorder in our sample is 8.38 per 10,000 person-years. This is similar to the same event-rate (8.8) reported for any autoimmune-disorder hospitalisation by Dube et al between ages 19 and 44 [35] and suggests that self-report is not leading to an over-reporting of medical history in our sample. Alongside this, the data collection paradigm used here prevented the incorporation of data on autoimmune disease symptom severity, which we have shown elsewhere can be predicted by low–i.e. depressed–mood in a more deeply phenotyped clinical cohort of rheumatoid arthritis patients [10].

A final limitation of this work is the reliance on self-report for indicators of both disease status and age at onset. Our confidence in the validity of our depression measure comes from several sources. The prevalence of depression in our sample at 18.3% is consistent with previous reports in high-income countries, for example, Bromet et al report 14.6% (SD = 0.2) [45]. The higher prevalence of depression in our sample may indicate that the repeated interviews minimise under-reporting of depression, as previously noted by Moffitt et al [46]. As expected, we see later onset for depression in males, and a higher prevalence in females [47,48]. We note that our sample has a later average age at onset (34.4 years) than reported elsewhere [2]; if this reflected a systematic bias in our study, it would mask any causative effect of depression on autoimmune disorder onset, as depression onset would be reported artificially late in individuals with both disorders. In sum, our depression measure appears to be reliable, and any possible systemic bias would not detract from our conclusions.

### Summary

We have replicated, and built on, previous studies in finding a significant association between major depressive disorder and the autoimmune disorders in an unselected, population-based sample. Furthermore, we have found significant evidence for depression temporally preceding autoimmune disorders in some patients, and vice versa. This suggests a causal effect of MDD on autoimmune disorder onset, perhaps via some depression-associated behaviour such as diet, or an environmental risk factor shared between the two phenotypes, such as cigarette smoking. Finally, we have used genetic data to demonstrate independent effects of autoimmune disorder status and MDD genetic risk scores on onset of depression.

## Supporting information

S1 File Assessing evidence for non-random dropout. (PDF)

S2 File Prevalence of Autoimmune Disorders. (PDF)

S3 File Determining age at onset of depression. (PDF)

S4 File Preparing Data for Rheumatoid Arthritis Polygenic Risk Score. (PDF)

S5 File Repeating Main Analysis with Cluster Robust Standard Errors. (PDF)
