# Supplementary material for: A bidirectional relationship between depression and the autoimmune disorders – New perspectives from the National Child Development Study
Source: PLoS One. 2017 Mar 6;12(3):e0173015. doi: 10.1371/journal.pone.0173015 (PMC5338810; doi:10.1371/journal.pone.0173015)
Supplement: S1 File — (PDF) [file pone.0173015.s001.pdf]

Supplementary 1: Assessing evidence for non-random dropout

We verified that attrition and data cleaning was not introducing bias into our sample. We obtained data at waves 0 – 7, harmonised based on identification number and performed the cleaning procedure as described in methods. In this sample, we investigated a number of variables as predictors of presence versus absence of individuals in a cleaned analytic sample using a multivariate logistic regression framework.

We selected a number of biomedical predictors recorded at wave 3 (age 16) in order to test whether attrition is independent with respect to our outcome measures – these are 'Seen a Psychiatrist or Psychologist for Depression', and some proxies for autoimmune disease – Diabetes, Psoriasis and Bowel Problems (the closest proxy to IBD available in the waves 0-3 data). We also selected a number of demographic variables – gender, general ability measured at age 11 and father's social class

	Estimate	Std. Error	z value	Pr(>|z|)
**Intercept**	-1.03	0.10	-9.86	6.26×10^{-23}
**General Ability Score**	0.02	0.00	18.88	1.68×10^{-79}
**Psoriasis**	-0.19	0.22	-0.86	0.39
**Diabetes**	0.38	0.54	0.71	0.48
**Bowel Problems**	-0.09	0.12	-0.78	0.44
**Seen a psychiatrist for Depression**	-0.05	0.33	-0.14	0.89
**Female**	0.22	0.04	6.06	1.38×10^{-9}
**Father's social class II**	0.03	0.09	0.33	0.74
**Father's social class III**	0.04	0.10	0.38	0.70
**Father's social class IV**	-0.08	0.09	-0.93	0.35
**Father's social class V**	-0.10	0.09	-1.03	0.30
**Father's social class VI**	-0.22	0.11	-1.97	0.05
**Father's social class VII**	-0.32	0.12	-2.80	0.01

Table S1: Predictors of attrition at wave 7 following data cleaning

As expected from many prior studies, low general ability score in childhood, female gender, and low paternal social class did predict attrition (Matthews et al 2006). Importantly for the current analyses, however, the medical disorders were not significant predictors of attrition. These results indicate that attrition is random with respect to our outcome measures.

Matthews F. E., Chatfield M. & Brayne C. (2006). An investigation of whether factors associated with short-term attrition change or persist over ten years: data from the Medical Research Council Cognitive Function and Ageing Study (MRC CFAS). *BMC Public Health*, 6:185.
