# Supplementary material for: A bidirectional relationship between depression and the autoimmune disorders – New perspectives from the National Child Development Study
Source: PLoS One. 2017 Mar 6;12(3):e0173015. doi: 10.1371/journal.pone.0173015 (PMC5338810; doi:10.1371/journal.pone.0173015)
Supplement: S2 File — (PDF) [file pone.0173015.s002.pdf]

Supplementary 2: Prevalence of Autoimmune Disorders

Below we have summarised the numbers of reported Autoimmune Disorders investigated in our sample, with data censored at age 46.

Autoimmune Disorder	Number of Cases	ICD-10 Code
Polyarthritis	163	M13
Rheumatoid Arthritis (Seronegative)	37	M06
Crohn's Disease	26	K50
Multiple Sclerosis	25	G35
Psoriasis	24	L40
Ulcerative Colitis	12	K51
Celiac Disease	11	K90
Type 1 Diabetes	5	E10
Sjogren Syndrome	4	M35
Pernicious Anemia	3	D51
Graves' Disease	2	E05
Pulmonary Fibrosis	2	J84
Hashimoto's	1	E06
Scleroderma	1	L94
Addison's Disease	0	E27
Dermatomyositis	0	M33
Granulomatosis	0	M31
Haemolitic Anaemia	0	D59
Lupus	0	M32
Myasthenia Gravis	0	G70
Myocarditis	0	I40
Rheumatoid Arthritis (Seropositive)	0	M05
Thrombocytopenia Purpura	0	D69
Vitiligo	0	L80

Table S2: Number of cases for each autoimmune disorder investigated, with ICD-10 code used for classification
