# Supplementary material for: A bidirectional relationship between depression and the autoimmune disorders – New perspectives from the National Child Development Study
Source: PLoS One. 2017 Mar 6;12(3):e0173015. doi: 10.1371/journal.pone.0173015 (PMC5338810; doi:10.1371/journal.pone.0173015)
Supplement: S3 File — (PDF) [file pone.0173015.s003.pdf]

Supplementary 3: Determining age at onset of depression

As outlined in the Methods section, we drew on reports from 3 study sweeps (waves 5, 6 and 7) to identify age at onset of depression. Not unexpectedly, we identified a small number of discrepancies in reporting across sweeps, and dealt with them as follows: Participants reporting a history of depression at wave 5 (age 33), but not at wave 6 (age 42) were excluded. Participants reporting depression at waves 5 and 6, but with reported ages at onset that differed by more than 10 years were removed. Participants with no reported onset at wave 5, but reported onset at wave 6 before age 23 were removed. Any individuals reporting age at onset for depression before age 7 were reassigned onset at age 7.

As a final step, we used 9 items from the Malaise Inventory, a tool to assess low mood (Rutter et al, 1970), to validate self-reported depression at wave 5 (Malaise items 2, 3, 5, 9, 12, 14, 16, 20, 21). We removed any cases who scored zero on the Malaise Inventory data collected at age 33, but who reported depression onset between ages 30 – 36.

Rutter M., Tizard J. & Whitmore K. (1970) The Malaise Inventory. *Education, Health and Behaviour*. Longman, London
