# Supplementary material for: A bidirectional relationship between depression and the autoimmune disorders – New perspectives from the National Child Development Study
Source: PLoS One. 2017 Mar 6;12(3):e0173015. doi: 10.1371/journal.pone.0173015 (PMC5338810; doi:10.1371/journal.pone.0173015)
Supplement: S4 File — (PDF) [file pone.0173015.s004.pdf]

Supplementary 4: Preparing Data for Rheumatoid Arthritis Polygenic Risk Score

In order to remove the effect of the WTCCC1 controls from the Okada et al (2014) GWAS, we first clean the WTCCC1 RA cases and controls using the following protocol. We first remove SNPs with minor allele frequency below 10% and Hardy-Weinberg Equilibrium *P*-value below 0.05, and individuals with missingness above 10%. We then remove SNPs with a genotyping rate below 99.5%. Finally, individuals with missingness above 1% on the remaining SNPs are removed. Following this protocol, 297,733 SNPs remain, and GWAS is performed on these cases and controls using logistic regression under an additive model.

After merging with the Okada et al GWAS, we remove SNPs with an average posterior call rate (as determined by Chiamo) > 99.9%, and ambiguous SNPs (i.e. A/T and C/G). This leads to a total of 178,239 SNPs.

We remove the effect of this GWAS using the formula below. This is a re-arrangement of the standard inverse variance weighted fixed effects meta-analysis formula, as presented elsewhere (Borenstein et al., 2009).

Where a full GWAS contains *only* effects from GWAS A and B, and:

β_{FULL} is effect size from full GWAS

β_{A} is effect size from GWAS A

β_{B} is effect size from GWAS B

SE_{FULL} is standard error from full GWAS

SE_{A} is standard error from GWAS A

SE_{B} is standard error from GWAS B

$$SE_B = \sqrt{\frac{{SE_{FULL}}^2}{1 - {SE_{FULL}}^2 / {SE_A}^2}}$$

$$\beta_B = {SE_B}^2 \left(\beta_{FULL} \left(\frac{1}{{SE_A}^2} + \frac{1}{{SE_B}^2} \right) - \frac{\beta_A}{{SE_A}^2} \right)$$

Borenstein, M., Hedges, L. V., Higgins, J., & Rothstein, H. R. (2009) *Introduction to Meta-Analysis* John Wiley & Sons, Ltd.
