# Supplementary material for: A bidirectional relationship between depression and the autoimmune disorders – New perspectives from the National Child Development Study
Source: PLoS One. 2017 Mar 6;12(3):e0173015. doi: 10.1371/journal.pone.0173015 (PMC5338810; doi:10.1371/journal.pone.0173015)
Supplement: S5 File — (PDF) [file pone.0173015.s005.pdf]

Supplementary 5: Repeating Main Analysis with Cluster Robust Standard Errors

In order to investigate the possible effect of longitudinal dependence of observations biasing our results, we repeat the main analyses here, accounting for any longitudinal dependence using cluster robust standard errors implemented in the survival package in R. The results are presented below as table S3, and show that omitting cluster robust standard errors in our main analysis biased estimates towards the null, therefore providing support for our conclusions. This is an expected result when calculating cluster robust standard errors for predictors with a negative intraclass correlation – this will be the case in our data, where individuals only contribute multiple rows if their disease status changes across the study period, thus inducing a negative intraclass correlation in these variables.

		Main Analysis		Using Cluster Robust Standard Errors	
		HR	*P*-value	HR	*P*-value
Predicting Depression Onset	Autoimmune Disorder (unadjusted)	1.39	3.71×10^{-3}	1.39	3.16×10^{-3}
	Autoimmune disorder (adjusted for Depression PRS)	1.31	0.046	1.31	0.044
	Depression PRS (adjusted for Autoimmune Disorder)	1.08	8.73×10^{-3}	1.08	8.17×10^{-3}
Predicting Autoimmune Disorder Onset	Depression (unadjusted)	1.40	9.55×10^{-3}	1.40	6.65×10^{-3}
	Depression (adjusted for Autoimmune Disorder PRS)	1.31	0.08	1.31	0.069
	Autoimmune Disorder PRS (adjusted for Depression)	1.15	0.03	1.15	0.041

Table S3: Comparison of using cluster robust standard errors with the main analysis presented above.
